# Survival, Morbidity, Growth and Developmental Delay for Babies Born Preterm in Low and Middle Income Countries – A Systematic Review of Outcomes Measured

**DOI:** 10.1371/journal.pone.0120566

**Published:** 2015-03-20

**Authors:** Melissa Gladstone, Clare Oliver, Nynke Van den Broek

**Affiliations:** 1 Department of Women and Children’s Health, Institute of Translational Medicine, University of Liverpool, Alder Hey NHS Foundation Trust, Liverpool, United Kingdom; 2 Centre for Maternal and Newborn Health, Liverpool School of Tropical Medicine, Liverpool, United Kingdom; Hôpital Robert Debré, FRANCE

## Abstract

**Background:**

Premature birth is the leading cause of neonatal death and second leading in children under 5. Information on outcomes of preterm babies surviving the early neonatal period is sparse although it is considered a major determinant of immediate and long-term morbidity.

**Methods:**

Systematic review of studies reporting outcomes for preterm babies in low and middle income settings was conducted using electronic databases, citation tracking, expert recommendations and “grey literature”. Reviewers screened titles, abstracts and articles. Data was extracted using inclusion and exclusion criteria, study site and facilities, assessment methods and outcomes of mortality, morbidity, growth and development. The Child Health Epidemiology Reference Group criteria (CHERG) were used to assess quality.

**Findings:**

Of 197 eligible publications, few (10.7%) were high quality (CHERG). The majority (83.3%) report on the outcome of a sample of preterm babies at time of birth or admission. Only 16.0% studies report population-based data using standardised mortality definitions. In 50.5% of studies, gestational age assessment method was unclear. Only 15.8% followed-up infants for 2 years or more. Growth was reported using standardised definitions but recommended morbidity definitions were rarely used. The criteria for assessment of neurodevelopmental outcomes was variable with few standardised tools - Bayley II was used in approximately 33% of studies, few studies undertook sensory assessments.

**Conclusions:**

To determine the relative contribution of preterm birth to the burden of disease in children and to inform the planning of healthcare interventions to address this burden, a renewed understanding of the assessment and documentation of outcomes for babies born preterm is needed. More studies assessing outcomes for preterm babies who survive the immediate newborn period are needed. More consistent use of data is vital with clear and aligned definitions of health outcomes in newborn (preterm or term) and intervention packages aimed to save lives and improve health.

## Introduction

The proportion of all deaths in children under-five years that occur in the first four weeks of life (neonatal death) has increased from 36% in 1990 to 43% in 2011 and 75% of neonatal deaths occur in the first week of life [1]. Of the estimated 7.6 million deaths in children under 5 years of age, an estimated 17%, are attributed to prematurity [1]. and approximately 35% are attributed to preterm birth (before 37 completed weeks or 259 days of pregnancy) [2], making prematurity the leading cause of neonatal death and the second leading cause of death in children under five years old [1].

Globally, around 10–11% of all births, or an estimated 15 million births per year, are estimated to be born preterm (before 37 weeks gestation) [3,4]. The incidence of preterm birth is around 10.6% in North America and 6.2% in Europe [3]. There are fewer reliable estimates from low and middle income settings because of uncertainty around assessment of gestational age and reliance on low birth weight as a proxy measure. However the incidence of preterm birth in these settings is considerably higher with estimates of between 15 and 24% in ultrasound dated population studies in African settings [5–8]. Although the reported rates of preterm birth are highest in sub Saharan Africa, the highest absolute number of preterm births occurs in Asia [4].

Preterm birth accounts for more than one million neonatal deaths per year. Information on the outcomes of babies born preterm but who survive the early neonatal period is very sparse [9,10]. Preterm birth is considered to be a major determinant of immediate as well as long term morbidity and is associated with growth and developmental delay. To be able to determine the relative contribution of preterm birth to the burden of disease in children under five years and to inform the planning of healthcare interventions to address this burden, a renewed understanding of the assessment and documentation of outcomes for babies born preterm is needed [11].

We undertook a systematic review of studies which report on outcomes for babies reported to be born preterm in low and middle income settings. For each study, we included the method of assessment of prematurity and outcomes including mortality, morbidity, growth and development.

## Methods

### Search strategy

A systematic search of all published literature using the following databases without language restrictions was conducted: Pubmed, Cochrane, Scopus, Ovid SP, Embase, WHO Regional Databases, CINAHL, American Psychological Association and Google. Search terms used included the MeSH terms and are shown below:

"Premature Birth" AND "Infant, Low Birth Weight" [MeSH] AND "Developing Countries" [Majr] AND “Outcome studies” OR “outcome assessment” OR “outcome measures” OR “treatment outcome” OR “outcome*” OR “endpoint”

We included the MeSH term of “low birth weight” as some papers used low birth weight as a proxy measure for prematurity. The search was repeated using a definitive list of 150 developing countries from the International Monetary Fund’s World Economic Outlook Report [12]. Snowball searching was done to identify additional key papers missed.

#### Study selection

Inclusion criteria for final review were primary research articles published after 1980 up to July 2014 which included 1) information on the follow up and any outcomes (survival, morbidity, growth and development) of prematurely born infant in any low and middle income country (according to World Bank criteria above) and 2) longitudinal cohort studies, randomised controlled trials and cross sectional studies and 3) full text articles for evaluation of all study components. Papers were excluded if they 1) had no information on outcomes of preterm birth (survival, morbidity, growth and development) 2) were inadvertently from a non-developing country setting 3) were systematic reviews with no direct data to inform our research question and 4) were case studies.

**Fig 1:**
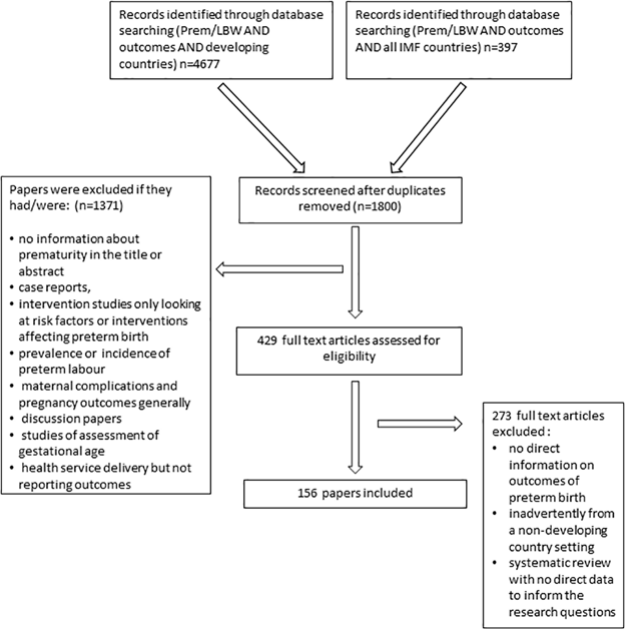
PRISMA Diagram.

We identified a total of 5321 titles through our searches. Two investigators screened these by title. Of 2023 eligible papers identified after duplicates had been removed, 902 full abstracts were then screened with 456 full text articles reviewed by the two investigators. Data items sought were information regarding recruitment, gestational age, methods of assessing and outcomes relating to mortality, morbidity, growth and development of infants (see S1 Dataset). Data was then extracted into a table which was piloted and reconfigured particularly relating the categorisation of place and time of recruitment, level of neonatal care which could be accessed and categorisation of mortality of infants (PNMR, NNMR, IMR). Where there were discrepancies in assessment, a third reviewer was consulted. In total, 197 publications were considered eligible for inclusion in this review (Fig. 1). We used the Child Health Epidemiology Reference Group (CHERG) criteria to assess the quality of articles [13].

## Results

A summary table of all included studies is provided (S1 Dataset).

### Quality of articles

Papers were assessed for quality using the CHERG criteria: the majority were of low quality (101/197, 51.3%) with 21 papers judged to be of high quality. Many were case series or small cohort studies particularly those from neonatal facilities in low income settings. For observational studies, if the research took into account all plausible confounders, then they would be upgraded.

### Study Setting

**Fig 2:**
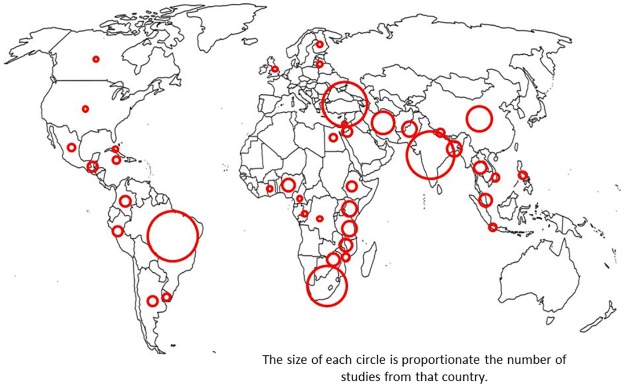
Geographical Distribution of Included Studies.

Equal numbers were from Asia (62/197, 31.5%) and Sub-Saharan Africa (56/197, 28.4%); 32 (16.2%) papers from Latin America and the West Indies, 43 (21.8%) from the Middle East and Eastern Europe and two reported on outcomes from multiple settings (Fig. 2). Out of studies from a single setting, 41/197 (20%) were from a low income setting, 48/197 (24%) from a low-middle income setting and just over half (103/197 (52%)) were from a higher middle income setting.

The majority of studies (164/197, 83.7%) report on outcomes of a sample of babies born preterm and recruited into the study at the time of birth or at the time of admission to a special care baby unit at a health facility. For the purpose of this review we have referred to these studies as ‘facility based studies’. Only 16.0% (31/197) of studies were from a community setting i.e. babies and/or mothers not recruited at time of visit to a health care facility but from among the general population usually at antenatal visits within a representative area serving a population. Two thirds of these studies (N = 22) came from low or low middle income settings. A total of 17.8% (35/197) studies reported population based data (sample size studied was all babies in the general population studied or a representative sample of babies from the general population) such as studies from Ghana [14,15], Nepal [16], Tanzania [17], Malawi [18] and Guatemala [19]. This included facility based studies from Brazil, China and Chile where it was reported that almost all births in the area would have taken place in a hospital [20–25]. In general, facility based studies tended to be small case control or cohort studies whereas community based studies were often larger population based observational cohort studies.

Just over half of the facility based studies reported on babies who had been admitted to a special/intensive care baby unit (114/197, 58.2%). This included 19 (9.7%) studies for babies from a special care unit with no ventilation (almost all from low or low middle income settings) and 63 (32.1%) from a neonatal intensive care unit with ventilator facilities. Papers reporting on outcomes from settings where neonatal care (including ventilation) was available were predominantly from the high middle income countries particularly the Middle East but included papers from Turkey, China, South Africa, Zimbabwe, India and Pakistan. In 32/114 (28.0%) of the studies reporting on babies admitted to a baby care unit, it was not clear what type of neonatal care was available. Often this was labelled as “neonatal intensive care” or ‘neonatal care facilities’ without specific description of content.

### Population

The numbers of infants included in the studies varied from 10–12 in one study of outcomes of preterm infants receiving continuous positive airway pressure (CPAP) in a neonatal intensive care unit [26] to 2.9 million infants born in a national study of neonatal survival conducted over 10 years in Chile [27]. The majority of papers (169/197, 86.2%) included in the review were cohort studies. Only three studies followed up on only the preterm infants born from a representative sample of the population [3,20,28]. More commonly, preterm infants were followed up as part of a subset of a larger study looking at neonatal outcomes of all infants (or those born low birth weight) in a community.

**Fig 3:**
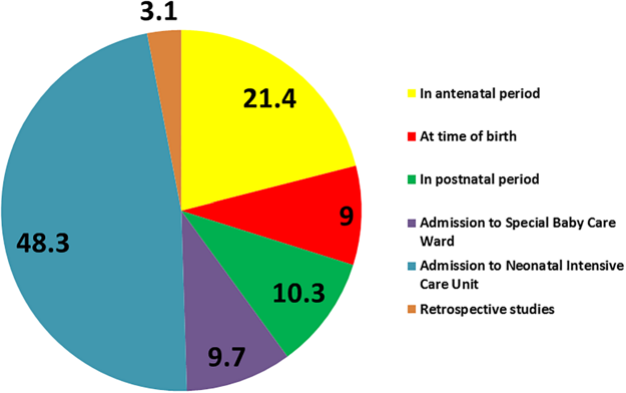
Setting and timing of recruitment for studies reporting on outcome of preterm birth in low and middle income settings (% studies).

In 20% of studies, the parent cohort was identified during the antenatal period. For other studies, preterm birth was determined at the time of birth, in the post natal period or at time of recruitment to a newborn care facility (Fig. 3).

In the majority of studies (114/197, 58%) outcomes are reported for babies admitted to a neonatal intensive care or special care unit (or unit named as a neonatal unit but with facilities not specified). Frequently, studies reported on a subgroup that had complications such as respiratory distress syndrome, intra-ventricular haemorrhage or retinopathy of prematurity.

The majority of studies were prospective but did not often include comparison groups and were unclear about the inclusion or exclusion of twin infants, a likely confounder in studies of outcomes of prematurity [29]. Just over a third (58/156, 37.2%) reported including twins.

### Length of follow up

Length of follow up varied from only 24–72 hours [30,31] to 15 years in the recent cohort studies from Brazil, India, Philippines, South Africa, Guatemala [32,33]. In half of all studies, the period of follow up was not clear. This includes a quarter (50/197, 25.5%) of studies with the period of follow up defined as “until discharge” from a health care facility and 23.9% (47/197) with no information at all provided about length of follow up. 15.8% (31/197) of studies followed up infants for 2 years or more. One recent study reports on the long term growth, blood glucose level and blood pressure outcomes in cohorts of children for over 15 years in Brazil, India, Philippines, South Africa and Guatemala, demonstrating some differences in long term height attainment of those born premature [32,33]. Two groups reported on long term cognitive and educational outcomes of infants born prematurely—the group in India [34] and that from the recent Stein paper [35].

#### Gestational age at birth

51.0% (100/197) of all studies provided information on the gestational age at birth with an equal spread across income settings. The majority of studies included all babies born at <37 weeks gestation but eight studies specifically targeted only late-preterm infants (34+^0^–36+^7^ weeks gestation) and studied outcomes including general morbidity [35–37], transcutaneous bilirubin [38], growth [30] neuro-behavioural outcomes [39] respiratory outcomes with antenatal steroids [40] or surfactant therapy [41]. A total of 47 studies reported only on babies born below 33 or 34 weeks with only a few of these concentrating on the extremely preterm [25,26,42].

A variety of criteria and methods of assessment were used to define gestational age at birth. In approximately half the studies (99/197, 50.5%), the method of assessment was unclear. 22% (44/197) of studies used a combination of methods with 7.2% (14/197) using a combination of Ballard/Dubowitz and last menstrual period (LMP), 15.3% (30/197) of studies used antenatal USS in combination with other methods—mainly LMP. USS dating has been used in some community settings in low income settings such as Malawi, Mozambique, Tanzania and Guatemala [6,8,28,42,43], studies originating from bigger teaching hospitals such as those from Brazil, Ghana, Zambia, India and South Africa [21,44,45] and studies where babies were recruited at health care facilities with neonatal units in both low and middle income settings such as Turkey, Malaysia, Oman and Congo [46–49]. Almost a third of studies used a single method of assessment to determine gestational age at birth. This consisted of: fundal height (two Malawi studies) [18,50], the Capurro score in two South American studies [19,51], Ballard score in six studies (6/197) [52–57] and Dubowitz score in six studies (6/197) [22,58–62]. LMP was the only information used to estimate gestational age at birth in 22 studies (22/197). Low birth weight was used as a proxy for prematurity in two studies [56,63].

Only eight studies consistently used USS dating of the pregnancy as the basis for calculation of gestational age at birth [54,63], one study specifically looking at Doppler flow in an Indian perinatal centre [64].

### Mortality

59% (116/197) of studies measured survival as an outcome with 49 of these (25%) using uniform definitions for describing mortality rates (perinatal, early neonatal, neonatal and infant) [65]. More than half of all studies (53.7%) 106/197 reported in-hospital mortality or survival during the period of follow up ‘post discharge’. 29 studies reported mortality but never specified a time period for which survival was assessed. In many studies, no denominator was reported and therefore it was unclear whether measurements were a rate (per 1000 live births) or a percentage of the babies included in the study. This makes comparison across studies difficult.

Out of 20 identified population based studies which reported on mortality rates, 19 reported mortality using the internationally agreed definitions for Perinatal Mortality Rate (PNMR), Neonatal Mortality Rate (NMR), Early and Late NMR. Data is summarised in Table 1. Almost all of these studies came from low and low middle income settings with a few population based studies from high middle income settings using systems for birth and death registration (studies from Chile and China). The others relied solely on follow up through the research studies, of mothers who had attended antenatal clinic. Most studies compared preterm births with term births. A few studies reported outcome separately by gestational age at birth [14,15,20,27,31,66,67] and some just look at outcomes of late preterm birth or compare this to term births [23,24,39,40].

**Table 1:** Mortality rates for babies born preterm and term with method of assessment of gestational age at birth.

Author	Country	Sample size	Assessment gestational age	Gestationalage range (wks)	PNMRPerinatal mortality rate		NMR (Neonatal mortality rate)					
							ENMR- Early neonatal mortality rate		Late neonatal mortality rate		Reported NMR (authors do not distinguish between early and late NNM)	
					Rate/1000	RR or OR	Rate/1000	RR or OR	Rate/1000	RR or OR	Rate/1000	RR or OR
**Slyker et al 2014(83)**	**Kenya**	**468 singleton pregnancies of HIV + women from ANC**	**LMP, Dubowitz and fundal height**	**All gest**								**Infant death incidence rate (IR) for preterm 7-fold higher than infants born at term (IR = 5.8 vs 0.81 per 1000 person-days, incidence rate ratio (IRR) = 7.1, 95%CI = 1.5–30, p = 0.008).**
**Ades et al 2013(84)**	**Uganda**	**351 live born infants**	**LMP and sonic biometry**	**All gest**								**<37 wks OR—12.7 (3.8–42.7). Increased GA assd with decreased OR of death—0.45 (0.2–0.9)**
**Welaga et al 2013(3)**	**Ghana**	**17751 births**	**LMP**	**All gest**			**Overall ENMR—16**				**<32 wks—62** **32–36–23** **>36 wks—19** **Overall NMR—24**	**<32 weeks-3.4 (2.66–4.32)** **<36 weeks—1.2 (0.95–1.54)**
**Engmann et al 2012(2)**	**Ghana**	**18,852 births (10.8% < 32 22.3%- 32–36 weeks)**	**LMP**	**All gest**	**<32 wks—186** **32–36 wks—33.7** **>36wks—16.4**	**<32 wks—13.8 (11.5–16.38)** **32–36 wks—2.1 (1.69–2.60)**	**<32 wks—37.3** **<36 wks—16.8** **>36 wks—12.1**	**<32 wks OR—3.16 (2.35–4.22)** **<36 wks OR—1.39 (1.05–1.84)**				
**Barros et al 2012(23)**	**Brazil**	**1577 births followed up**	**LMP**	**All gest**							**<34 wks—168** **34–36 wks—19** **<37 wks—11** **38 wks—8** **39–41 wks—4** **Overall NMR (all gest)– 12**	**<34 wks—34.4(21.6–54.8)** **34–36 wks—3.4 (1.8–6.6)** **<37 wks—2.7 (1.3–5.6)** **38 wks—2.0 (1.1–3.5)** **39–41–1.0** *Adjusted RR only provided
**Schmiegelowl 2012 (85)**	**Tanzania**	**872 women from ANC**	**USS**	**<37**	**52 (all gestations)**	**Crude OR: <37 wks: 23.07 (10.8–49.12)** **Adjusted OR: <37 wks** **14.47 (3.2–64.8)**						
**Graner et al 2010 (86)**	**Vietnam**	**5521 births**	**Unclear**	**< 37**	**25(all gestations)**	**Adjusted OR: <37 wks 9.15 (CI 4.7–17.8)**					**11.6 (all gestations)**	**Adjusted OR <37 wks** **7.83 (4.1–14.9)**
Pileggi et al 2010 (87)*	**Brazil**	**15,377 births 19 hospitals**	**Unclear**	**<37**			**<30 wks-430** **<37 wks- 71**					
**Engmann 2009 (88)**	**DR Congo**	**7959 births—women from ANC**	**LMP**	**24 - <37**	**<37wks- 738** **>37 wks—38**	**OR (multivariate analysis) 71.1 (47.6–106.3)**	**<37 wks—573** **>37 wks—19.1**			**OR (multivariate analysis) 68.8 (39.9–118.6)**		
**Gonzalez et al 2006 (35)**	**Chile**	**29.4 million births (national database)**	**Unclear**	**All**							*Rates given for 1990–2000 **27 wks: 405–276** **30 wks: 231–142.6** **34 wks: 59–29.4** **37 wks: 59–29.4** **38 wks: 4–2.4** **41 wks: 2.7–2** **Overall NMR (all gest): 8.3–5.7**	
Barros et al 2005 *(89)	**Brazil**	**6011 (1984)5304 (1993)2427 (2004)Births**	**LMP**	**<37**						**1982** **<34 wks—490** **>34–36+ wks-33** **1993** **<34 wks 153** **34–36+ wks-13**		
**Van den Broek et al 2005 (90)**	**Malawi**	**449 babies—ANC in Malawian districts**	**USS**	**24–37**	**21.7% prems died vs 3.4% term** **>32–37 wks 6.9% vs 3.4% died**	**Risk ratio 6.32 (3.2–12.45) term vs preterm**						
**Osman et al 2001 (91)**	**Mozambique**	**908 women—ANC**	**USS**	**21 - <37**		**<37 wks Adj OR 8.48 (3.4–20.9)** **OR—23.47 (11.5–47.9)**						
**Kulmala et al 2000 (6)**	**Malawi**	**813 births (all women in ANC)**	**Fundal height**	**<38**	**65.3 (all gestations)**	**If <38 wks** **Adj OR 9.6 (4.4–21) p<0.001**					**37- (all gestations)**	**If <38 wks adj OR 11 (3.6–32.4) p<0.001**
Xu et al 1998 (92)*	**China**	**9207 births from birth records**		**>28**	**Preterm < 2.5 kg—306.9** **Preterm >2.5 kg-26.3**							
**Kapoor et al 1996 (93)**	**India**	**966 births (live and still)**	**Unclear**	**<37**	**<37 wks—273**	**RR 1.95 (1.36–2.8)**						
**Schreiber et al 1994 (94)**	**Guatemala**	**120/120 cases & controls** **From civil registry**	**Unclear**	**Not clear**		**OR of 17.1 (5–59.3) Coeff-2.8(SE 0.6)**						
Gray 1991 (95)*	**Brazil**	**11,171 live births**	**Capurro score**	**Proxy:b-wt <2.5kg**			**591 preterm SGA** **318 preterm AGA** **25 (all gest)**	**OR—preterm/LBW—45.1 (32.4–62.9)** **Adj OR preterm LBW 52.1(33.8–80.4)**				
Zhang et al 1991 (22)*	**China/ Shanghai**	**1134—every birth in Shanghai**	**Not clear**	**>28**	**28–36 wks—137.7** **37–41 wks—9.2#** **Overall all gest—15**				**28–36 wks—70.2** **37–41 wks—3.9** **Overall all gest- 6.9**			

RR—Relative risk OR—odds ratio

All studies report an increased mortality rate among babies born preterm or report prematurity as the leading cause of death in neonates with lower gestational ages associated with increased mortality.

### Morbidity

There is currently no agreed set of criteria to define neonatal or infant morbidity. In the included studies, morbidity was defined in a variety of ways. The Simplified Newborn Illness Severity and Mortality Risk Score II (SNAPP II) was used in two studies from tertiary neonatal units in Brazil [68,69].

Measures of morbidity used particularly in community based studies included; need for hospitalisation and prevalence of wheezing and pneumonia [23], number of routine and additional clinic visits in the post natal period [19] or a pictorial diary with specific measurements of diarrhoea and ways of measuring body temperature and respiratory rate [17]. Studies with an emphasis on facility based care used proxy measures for morbidity such as need for transfer to NICU [49], length of time in NICU [70,71,72] time spent in oxygen [73] and time spent on ventilator [41,74–76], number of routine and additional clinic visits in the post natal period [19].

Specific morbidities relating to preterm in neonatal intensive care units (mainly in the higher middle income countries) used well defined criteria in some cases. For example intra-ventricular haemorrhage [77,78] was defined using the Levene staging system [79] or the Papile classification [80–82]. Studies looking at necrotising enterocolitis [53,81,83–88] used the Bells (or modified Bell’s) criteria [89] and some studies [77] using the Gidieon classification for respiratory distress syndrome [90] or a classification for, broncho-pulmonary dysplasia [52,91–96]. Some studies looking at retinopathy of prematurity [75,76,92–95,98–100] used the International Classification of Retinopathy of Prematurity (ICROP) [75,97,99–101]. These well-defined outcomes tools were not however systematically used across all studies in neonatal units which reported these morbidities.

The majority of these facility based studies report an increased risk of morbidity in preterm infants compared to babies born at term. This includes increased risk of respiratory distress syndrome (RDS) [52,59,102,103], broncho-pulmonary dysplasia (BPD) [68,97,104–108], retinopathy of prematurity (ROP) [73,101,106,109] intraventricular haemorrhage (IVH) [72,110], periventricular leukomalacia (PVL) [111] and cerebral palsy [55,58,112]. Studies are however not comparable due to differences in diagnostic approach and level of care available in the different settings. In addition, lack of information on the range of gestational age at birth even in hospital populations studied makes comparison difficult. Some studies specifically looked at morbidity in the late preterm group and report an increased risk of morbidity including hyperbilirubinaemia, sepsis, wheezing and hospital admissions post discharge [23,30,35–37,39,103,113].

### Growth

Less than 50% of all included studies report on growth outcomes. Where growth was reported, it was in a mixture of both low and middle income studies, this is usually done using standard internationally agreed methods and includes measurement of head circumference, height, weight (and in combination) using CDC or WHO standard growth curves for comparison.

Overall studies report that babies born preterm do not meet the same growth targets as babies born at term, continue to remain below the standard growth curve and demonstrate reduced ability for catch up growth. This is particularly well documented in the larger prospective community studies—almost all which had a follow up period of two years—from Malawi [28], Tanzania [114], India [115], Pakistan (3 year follow up) [116] China [117] and Brazil [23] as well as the more recent longer term cohort studies from India, Philippines, Brazil, Guatemala and South Africa [33]. This demonstrated lack of complete catch up growth at 15 years in those born premature or born at term but who were small for gestational age. It also demonstrated how those who did have catch up growth in the post natal period did make gains in height and schooling regardless of birth status. Interestingly, the studies documenting growth outcomes for babies who had received care in a well-equipped health care facility Cooper in South Africa [55] and Ho in Hong Kong [70] reported that there was less evidence of differences in growth between babies born preterm and term. In Kenya, where neonatal special care facilities are much more limited, only 20–28% of infants born preterm reached the lower limit of normal growth by term [118].

### Development

In total, only 38/197 (19.4%) of the studies found reported on development and/or neurological outcomes of babies born preterm. Studies came equally from low, low middle and high middle income settings. 5 of these studies were from neonatal intensive care units in countries such as China, South Africa and Turkey with some of these studies examining specific cohorts of children such as those with periventricular echogenicities [46], cord pH at birth [119], the use of ferritin [120] or absent end diastolic flow [83]. Only five studies were population based: Malawi [28], Pakistan [116], Ethiopia [121], Guatemala [43] and a recent large study in Nepal [16]. The majority of other studies were from babies who had been admitted to a neonatal unit including India [34,58,122–125], Bangladesh [126], Kenya [112] and Taiwan were developmental care was provided [97]. Outcome measures include a wide range of developmental cognitive educational or neurological assessments (Table 2). Some studies conducted comprehensive neurological and developmental assessments as well as vision and hearing testing [55,93,121,126,127]. Only a few of these studies clarified criteria for neurological impairment or cerebral palsy [93,112]. 82% (31/38) of those studies assessing development or cognition used the Bayley Scales of Infant Development II or III with two studies using an adapted Indian version. Other developmental tools used were the Griffiths Mental Development Scales [54,70], the Denver II [22] or the Gesell Developmental Scales [117] (some adapted or validated for a specific setting). Some used tools created for a specific region such as the Malawi Developmental Assessment Tool [128] or the Ankara Developmental Screening Inventory [129]. Some used specific tools for one area of development or ability such as the Peabody Developmental Motor Scale, the Movement ABC or the Reynell Developmental Language Scales. Cognitive measures which were used varied and included the WISC III, WISC-R Bender Gestalt and Human figure drawing tests, WAIS, Kaufman ABC and the Stanford Binet. Behavioural measures were used in a minority of studies and included the Achenbach questionnaires and Raval’s scales of social maturity. Neurological assessments were conducted in a few studies with some using specific classification systems such as those by Costello [130], Robertson [131], Saigal and Rosenbaum [132] or Amiel-Tison and Gosselin [133].

**Table 2:** Assessment of neuro-development for babies born preterm in low and middle income countries.

Developmental outcome tools used	Cognitive outcome measures used	Neurological and sensory assessments	Specific outcome measures eg. Speech and language or OT	Classification and identification of specific disability
Denver Development Screening TestDevelopment Screening Inventory Denver II [22] Gesell developmental scales for 0–3 yrs revised by Chinese Pediatric Association and Beijing Mental Development Cooperative Group [117] Bayley Scales of Infant Development (Indian Norms) Bayley Scales of Infant Development II (BSID II) Peabody Developmental Motor Scale Alberta Infant Motor Scale Development delay determined by Dorothy Egan's Model Malawi Developmental Assessment Tool [128] Griffith’s Developmental Assessment Scales (also for Columbia) [54,70] Ankara Developmental Screening Inventory [129] 17 milestone gross motor development scale (Jahari) Munich functional developmental diagnostics: gross motor and fine motor skills, perception, active speech, comprehension of speech, age of social interaction and independence [96]	Stanford Binet Intelligence scales Bender Gestalt Test and the Human figure drawing test “School progress reports/performance” Weschler's inteligence scale (WISC-Revised) Wide-range Achievement Test (WRAT) Universal Non Verbal Intelligence Test (UNIT) Stroop and Backward Digit span (Weschler)	“Assessment in high risk clinic, hearing and vision assessment” “Hearing and ophthalmic assessment” Tympanomtetry and free field audiometry with a pure tone audiometer “Examined for neurodevelopmental impairments and disabilities. If problems, EEG or USS used to investigate” Spontaneous movements (Prechtls qualitative assessment of general movements) “hearing screening, vision (squint), ROP, Assessed for CP and IVH (USS)” Hammersmith neonatal neurological assessment Neonatal Neurobehavioural Exam—Chinese (NNE-C) BSEAs—Brain stem evoked auditory potentials Neurobehavioural Asseement of the Preterm Infant (NAPI) [99]	Combined Amiel Tison [133] Methods Raval's Scale of Social Maturity OT assessment “Social and environmental assessment” Draw-a-person Screening Procedure for Emotional Disturbance (DAP-SPED) Movement Assessment Battery for Children (ABC) Finger tapping test Early language milestone (ELM) scale Amiel Tison & Gosselin [133] method for describing specific upper motor neurone abnormalities such as muscle tone and reflexes Achenbach's Child Behaviour Check List	Neurodevelopmental status assessed by physio providing neurodevelopmental score (NDS) to child. Gross Motor Function (GMF) and GMFCS Functional disability (physical and cognitive development) determined by Saigal and Rosenbaum's method [132] Cerebral palsy defined as 'presence of abnormal muscle tone or power in one or more limbs or the trunk. CP defined as a “nonoprogressive CNS disorder with abnormal muscle tone with classification system for mild moderate or severe CP. Bilateral severe hearing loss—permanent hearing loss that required amplification in both ears. Bilateral blindness—absence of functional vision in either eye. Neuro-developmental impairment—mod to severe CP, an MDI or PDI of < 70, bilateral deafness or bilateral blindness. “Profound impairment”—MDI < 50 or GMFCS level 4 or 5. “Minimal impairment” defined as an MDI or PDI score 70–84 & not having moderate to severe cerebral palsy, bilateral severe hearing loss or blindness.

Although studies varied in terms of outcomes and are difficult to compare, generally there were poorer developmental outcomes in babies born preterm compared to term.

## Discussion

Prematurity is the leading cause of neonatal mortality worldwide and one of the limiting factors for achieving a two thirds reduction in under five mortality rate between 1990 and 2015 (Millennium Development Goal 4) [134].

Alongside this, reports of a very high incidence of prematurity from reliable studies in low and middle income settings (with good estimates of gestational age at delivery and representative population samples) are now emerging.

This review identified almost 200 studies which report on outcome for babies born preterm. Studies represent a good geographical spread. Very few studies are from a community setting or provide population based data; most studies report outcomes for babies born in a health care facility and/or accessing health care because of identified health problems associated with preterm birth. With an estimated 47% of babies born with skilled birth attendance in low income countries and 60% in lower middle income countries [1] this means that there is currently no information about the majority of babies born preterm who have no access to health care and for whom outcomes might be worse than those reported in this review. Even for those where health care was available, the outcome for babies born preterm will depend on the availability and uptake of newborn care. In almost 30% of all studies reporting on babies who had received care at a ‘baby care unit’ there was no information about the level of clinical care and in many more cases, the information was inferred by reading the article carefully rather than it being reported clearly within the text.

We noted considerable variation in recruitment of participants, location of study, method of assessing gestational age and whether still births were included in the figures. Another key determinant of survival for babies born preterm is gestational age at time of birth. Only 51% of studies reported on the gestational ages of the infants. Furthermore the actual method of assessment was not clear in fifty percent of the studies. Gestational age, where reported, was most frequently estimated only after birth via assessment of appearance of the baby with/ without taking into account recall of the date of the last menstrual period. Only eight studies consistently used ultrasound scan dating.

For studies reporting ‘mortality rates’ both the denominator and nominator were not clear and did not fit with standard definitions, for example, perinatal, early neonatal, late neonatal or infant mortality. After extensive examination of the data it became clear that comparison of these data and conduct of a meta-analysis is currently not possible. A recent paper published by Katz includes a meta-analysis from original datasets obtained specifically for this purpose. This study does provide pooled overall relative risks for preterm neonatal mortality at 6.82 and 2.50 for post neonatal mortality with higher rates for those born both preterm and small for gestational age (SGA) 15.42 [135]. Similarly in studies we identified in this systematic review mortality rates are consistently higher in preterm births than in term births.

There are no currently agreed standard criteria to capture neonatal morbidity. In this systematic review, key morbidities in facilities in low and middle income settings do not seem to be dissimilar from those seen in high income settings [136]. Tools used for measuring neonatal morbidity in neonatal units such as the SNAPPE II [137] uses physiological indicators which cannot be feasibly obtained in most low income settings. Some standard definitions for morbidities such as intraventricular haemorrhage or retinopathy or prematurity are present but are not useful in community settings where little facilities are present for diagnoses. Some studies used criteria for assessing sepsis. None of these were defined according to internationally recognised criteria. However, it is likely that some are similar to that defined in the Young Infants Clinical Signs Study [138] which could be used more frequently. In contrast, growth was assessed using comparable criteria across the majority of studies. Similar to high income settings, growth falters with inadequate feeding in the preterm period.

The assessment of neuro-developmental outcomes was extremely variable with a variety of tools measuring a range of domains of neurodevelopment including general development, specific cognitive outcomes, physical examination measurements or specific sensory outcomes.

Apart from growth as an outcome, there are no standard definitions for criteria for morbidity and development and even though there are standard definitions for mortality, these are often not used.

**Fig 4:**
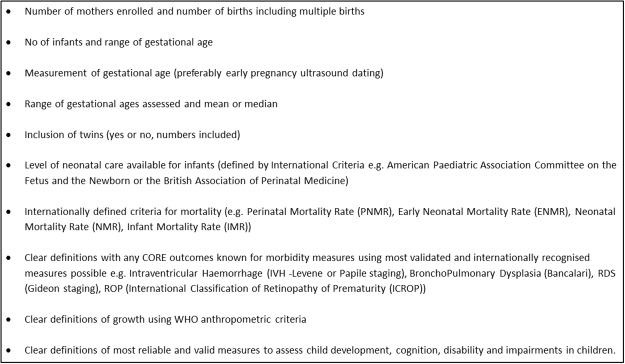
Recommended reported measures for studies on neonatal outcomes.

This review has highlighted the need for more robust studies assessing the outcomes for babies born preterm but who survive the immediate newborn period. It is vital that more consistent use of data is encouraged with clear and aligned definitions of both health outcomes in the newborn (preterm or term) and the intervention packages aimed to save lives and improve health. Methods of gestational age assessment, care packages available and outcomes to be assessed will need to be clearly defined and standardised to truly measure the burden of disease associated with preterm birth as well as to assess the effect of interventions to prevent or reduce morbidity and developmental delay in babies who survive. Similar to the call for core outcome indicators for trials [139] and the CROWN initiative [140] asking for core outcome measures in Women’s Health, we would recommend the development and adaptation of an agreed framework and indicators for the reporting of outcomes following preterm birth (Fig. 4). With the global burden of disease pertaining not only to mortality but also to morbidity, it is important that indicators assess these outcomes as well. Without this we will continue to lack the evidence needed to decide which interventions are most effective to improve outcomes for the large number of preterm babies born in low and middle income countries.

## Supporting Information

S1 Dataset Complete list of full text articles searched with study characteristics. (XLS)
